# Surgical site infections following caesarean operations at a Jordanian teaching hospital: Frequency and implicated factors

**DOI:** 10.1038/s41598-017-12431-2

**Published:** 2017-09-22

**Authors:** Mariam Hantash Abdel Jalil, Khawla Abu Hammour, Mervat Alsous, Wedad Awad, Rand Hadadden, Faris Bakri, Kamil Fram

**Affiliations:** 1 Department of Biopharmaceutics and Clinical Pharmacy, Faculty of Pharmacy, University of Jordan, Amman, Jordan; 2 Department of Clinical Pharmacy and Therapeutics, Faculty of Pharmacy, Applied Science Private University, Amman, Jordan; 3 Department of Medicine-Division of Infectious Diseases, University of Jordan, Jordan University Hospital, Amman, Jordan; 4 Infectious Diseases and Vaccine Centre, University of Jordan, Amman, Jordan; 5 Department of Obstetrics and Gynaecology, University of Jordan, Jordan University Hospital, Amman, Jordan

## Abstract

Surgical site infections (SSIs) following caesarean surgeries are common. The present study aimed to evaluate the frequency of SSIs following caesareans at Jordan University Hospital during the 30 postoperative days and to identify factors associated with increased SSIs risk. Data regarding the occurrence of SSIs were collected both prospectively via follow-up phone calls and retrospectively via reviewing wound culture results and clinical notes. SSI cases were subsequently determined utilizing predefined criteria. Data relating to possible risk factors of SSIs were collected from patient interviews and hospital records. Risk factors for SSIs were identified via logistic regression. A high rate of SSIs (14.4%) was detected; implicated factors included body mass index ≥36 kg/m^2^ prior to pregnancy odds ratio (OR) 3.8, 95% confidence interval (95% CI) 1.6–9.4, hospital stay longer than 3.5 days OR 2.3, 95% CI 1.4-3.6, having the operation at a gestational age greater than 40 weeks OR 2.2, 95% CI 1.3-3.9. Receiving a higher weight-adjusted dose of the prophylactic antibiotic cefazolin was associated with lower SSIs risk OR 0.967, 95% CI 0.94-0.99.﻿﻿﻿In conclusion﻿, a﻿ high rate of SSIs following caesareans was detected, and modifiable risk factors of SSIs should be incorporated into targeted policies aiming to reduce the rate of SSIs.

## Introduction

Based on world health organization (WHO) data, one in three patients receiving a surgical procedure in low-income/middle-income countries is predisposed to be affected by surgical site infections (SSIs)^1^. Of the different types of surgical wound infections, caesarean wound infections are of particular interest, as they are of the main infectious caesarean complications and are associated with elevated health care costs and maternal morbidity^2^. Another factor that emphasizes the clinical significance of such infections is the rising worldwide rates of caesarean deliveries^3^; this growing rate predicts a parallel increase in caesarean surgical site infection cases. Jordan, a middle-eastern developing country, is no exception to these rising rates, as the Jordanian caesarean rate increased from 18.2% in 2002 to 30.3% in 2012^4^.

The reported rate of SSIs varies considerably amongst the studies conducted at different centres around the world (2.1–48.0%)^5–8^, depending on differences in methodological issues such as the length of follow-up, the use of prophylactic antibiotics, and various patient-specific and surgery-related factors. To detect a notable decrease in the rate of SSIs at any centre, the rate of SSIs needs to be determined accurately, and risk factors associated with SSIs need to be investigated. This would allow policymakers to design targeted plans to eliminate modifiable risk factors of SSIs.

There are few Jordanian studies relating to SSIs following caesareans. Kaplan *et al*. reported that the rate of wound infections during hospital admission in 1,319 caesareans conducted between 1998 and 2002 was 8.1%^9^. In fact, this rate underestimates the true prevalence of caesarean wound infections, as the onset of such infections is usually detected post-discharge. For example, in a large-scale study conducted in England only 5.3% of the total infections were detected during the initial hospital stay^10^, while the rest were detected following discharge during the 30 postoperative days. This result pinpoints the importance of post-discharge surveillance systems in identifying SSI cases.

Considering the fact that there is no active post-discharge surveillance policy for monitoring SSIs implemented by our hospital’s infection control team, limited data are available relating to the development of SSIs during the first postoperative month. The present study was designed to evaluate the frequency of surgical site infection during the initial 30 postoperative days, utilizing multiple surveillance methods. Furthermore, this study aimed to identify factors associated with an increased risk of SSIs.

## Methodology

### Study location and ethical approval

The present research was conducted at the Jordan University Hospital (JUH) - Department of Obstetrics and Gynaecology for a period of eleven months from July 2015 to May 2016. The protocol of the study was approved by the Department of Biopharmaceutics and Clinical Pharmacy of the University of Jordan and then by the institutional review board of the JUH. This study was conducted in accordance with JUH’s ethical policy for preserving the rights of patients involved in research studies (policy number: Adm P021).

### Patient recruitment and baseline data collection

**Figure 1:**
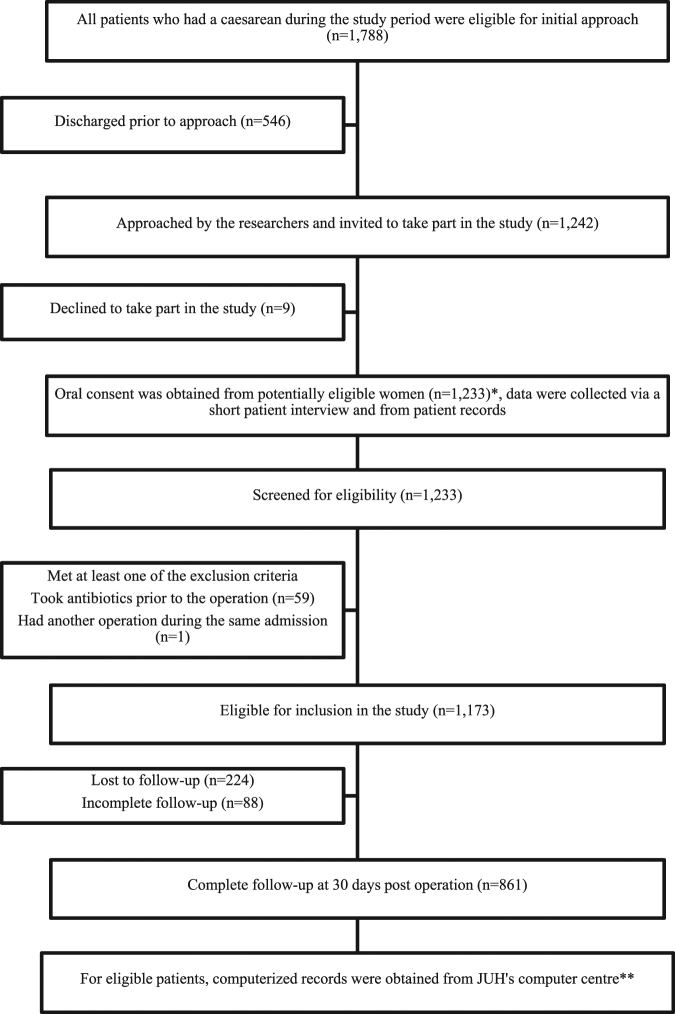
Flow diagram of patient enrolment, follow-up and data collection process. *To assure completeness of the collected data >95% of patient records were re-checked throughout the study period, once the files were archived. **Computerized patient records were also retrieved from the hospital’s computer centre; these records included patient-specific data in addition to clinical data such as dispensed medications and culture results.

All women who underwent a caesarean operation at JUH were eligible for the study unless they fulfilled one of the following criteria: a. Women receiving multiple doses of antibiotics for an established infection during their preoperative hospital stay; b. Women who had another operation during the same admission requiring prophylactic antibiotics. Patient enrolment in the study and follow-up in addition to the data collection process is summarized in Fig. 1. After obtaining oral consent from potentially eligible women, data relating to variables that could potentially be associated with SSIs were collected directly via a short patient interview and from patient records. These variables included patient-specific factors such as age, body mass index (BMI) prior to pregnancy, appropriateness of weight gain during pregnancy^11^ and smoking status. Surgery-related factors were also included such as type of anaesthesia, type of surgery (planned vs. emergent) and duration of surgery. Finally, factors related to the practice of administering prophylactic antibiotics prior to the operation were also investigated. The infection control team at JUH adapted the American Society of Health-System Pharmacists (ASHP) clinical practice guidelines for antimicrobial prophylaxis^12^ as their surgical antibiotic prophylaxis protocol. For caesareans in absence of β-lactam allergy, the ASHP recommends administering a single 2-g cefazolin dose to women weighing less than 120-kg, within a period of 60 minutes prior to the incision. In the present study, we examined the impact of non-compliance with two components of the ASHP guidelines, which are the dose of cefazolin (ASHP recommended 2-g dose vs. 1-g dose) and the duration of antibiotic administration (single preoperative dose vs. multiple doses) in relation with SSIs.

### Determination of surgical site infection cases

Multiple approaches were utilized to collect data relating to the development of SSIs in the abdominal wound during the 30 postoperative days. First, all available wound culture results for the included patients were retrieved from the computer centre of the hospital and positive cultures were recorded; at JUH, cultures are usually ordered by the attending physician, as part of routine care, when upon clinical examination of the wound signs of infection are noted. Second, all available computerized clinical notes from outpatient visits were also retrieved, and patients diagnosed with SSIs by the hospital physicians during the study period were identified. Finally, all patients who agreed to be re-contacted were called 30 days following the operation. In total, there were three phone call attempts for each patient, and patients who did not answer the first time were called two additional times on two different days. Patients who did not answer after three phone calls were not contacted again. A summary of questions asked during the phone interview is shown in Supplementary information Table S1, detailed answers were recorded directly on an a priori-designed Excel sheet. This approach allowed the research team to identify cases of SSIs that would otherwise go undetected simply because the patient chose to seek medical advice elsewhere following discharge.

Each phone call record was transcribed by three different researchers into an analysable format via a custom made Excel form; then, the main researcher checked the results of the three transcriptions and resolved any disagreement.

The data obtained from the three methods were pooled together and patients were allocated to the surgical site infection group based on fulfilling one the following criteria adapted from the Centres for Disease Control and Prevention (CDC) definition of SSIs^13,14^:

- A surgical site infection was diagnosed by the attending physician at the hospital’s outpatient clinics during the first 30 days postoperation.
- Results of culture indicate the presence of microorganisms.
- All patients who agreed to be re-contacted were called 30 days following the operation. Women were then allocated to the SSI group if they met one of the criteria for identifying patient reported surgical site infection adapted with modifications from Wloch *et al*.^10^, as shown in Supplementary Table S2.

### Sample size calculations and data analysis

The minimum required sample size for the present analysis was 323 patients, based on a maximum annual population size of 2,000 caesareans, 5% margin of error, 95% confidence level, and 50% response distribution as calculated by the Raosoft sample size calculator (http://www.raosoft.com/samplesize.html). An increase in sample size was desired because it is widely accepted that at least 10 events per predictor are required in a regression analysis to increase the stability of estimates^15,16^. All data were coded into Excel and then analysed via SPSS version 22 (IBM SPSS Inc., Armonk, NY, USA).

Correlations between categorical covariates and SSIs were explored utilizing a chi-square test; a Mann-Whitney U-test was utilized for the analysis of continuous predictors, as results of the Shapiro–Wilk test indicated that all the continuous variables investigated were not distributed in a normal fashion. Covariates with p < 0.05 were further investigated via binary logistic regression, and a p-value of less than 0.05 was considered statistically significant.

## Results

### Study participants

During the study period, a total of 1,233 women were approached and  were initially recruited into the study after obtaining oral consent, and 1,222 (99.1%) of these women agreed to be re-contacted via a phone call one month after the operation and provided the researchers with their personal phone number. The overall rate of follow-up via phone calls was 72.0%. Reasons for loss of follow-up included wrong phone number, disconnected, unreachable number or travelling abroad at the time of follow-up. Phone calls were in general brief, lasting only a few minutes. Sixty women met one of the previously specified exclusion criteria. Of the remaining 1,173 women, a total of 312 women were excluded due to the absence of follow-up or incomplete follow-up (Fig. 1). The patient-specific, procedure-specific and practice-specific variables investigated in the present study for their association with SSIs in both the included and the excluded women are shown in Supplementary Table S3. As noted in the table, no significant difference was observed in any investigated variable between the included and the excluded women.

The overall rate of readmission to JUH (regardless of the cause) during the initial postpartum period was low; less than 2.0% of the 1,173 women were readmitted.

### Frequency of SSIs

Utilizing the multiple approaches described previously, a total of 861 participants were followed-up for the entire 30 postoperative days. Wound infections were identified in 124 cases, constituting 14.4% of the included participants. The detected infections were identified by the physicians during outpatient visits or through positive cultures in 37.1% of infected patients, while the remaining infections were identified only via the phone interviews. When the infections were detected in the hospital (predominantly through outpatient clinics), they were identified on median postoperative day 10.8 (range, 3.3–26.6). Of the 312 women who were lost to follow-up, there were 83 women who did not answer phone calls and showed no signs of infection at last follow-up, which was on median postoperative day 9.6 (range, 5.4–21.3), these women were excluded from the infection analysis as it was not possible to determine whether they had developed an infection after the last follow-up. Furthermore, five patients with no follow-up of any type during the 30 postoperative days who visited the outpatient clinic or who had negative culture results after 30 days were also excluded from the analysis, as no data were available regarding SSIs during the 30 postoperative days. Culture results for the first 30 postoperative days were available for 41 of the infected patients. More than 10 types of pathogens were identified in the culture results. In 22 cases, more than one organism was identified in the culture results of the wound; therefore, the total number of infection-causing microorganisms isolated from the cultures was 67. The frequency of identified microorganisms is shown in Table 1.

**Table 1:** Frequency of the microorganisms isolated from wound cultures.

Type of microorganism	Frequency (%)*
***Enterococcus*** **species**	**22.4**
**Gram negative bacilli**-***Enterobacteriaceae***	**28.3**
*Escherichia coli*	13.4
*Enterobacter* species	3.0
*Klebsiella* species	10.4
*Proteus* species	1.5
***Pseudomonas aeruginosa***	**1.5**
***Staphylococcus*** **species**	**37.3**
***Streptococcus*** **species**	**6.0**
*Streptococcus pyogenes* -Group A	1.5
*Streptococcus agalactiae* -Group B	3.0
*Streptococcus bovis* - Group D	1.5
**Other**	**4.5**

*Calculated based on a total of 67 identified pathogens; in 22 wounds more than one pathogen was isolated.

### Factors associated with surgical site infection

Table 2 shows a summary of variables investigated for association with SSIs in the included women. A full list of tested variables in both the included and the excluded women is available in the Supplementary information Table S3. During the chi-square analysis, obesity prior to pregnancy (as categorized by the WHO BMI classification system) showed a significant association with infection. Infection rates increased from 10.6% in normal weight women to 23.3% and 33.3% in class-II and class-III obesity, respectively. Further in-depth analysis revealed that the strongest correlation was with a BMI ≥36.0; therefore, this cutoff point was used for the multiple regression analysis. The 2-g dose was only marginally associated with a lower rate of SSIs compared with the 1-g dose (p = 0.065, chi-square analysis). However, when the dose of cefazolin was standardized by each patient’s term weight, the median weight-adjusted dose of cefazolin was significantly lower in women who developed a surgical site infection, as shown in Table 2. Administering multiple antibiotics following caesarean during hospitalization did not reduce the risk of caesarean infection; patients receiving cefazolin preoperatively had similar rates of infection compared to patients receiving cefazolin as a prophylactic treatment preoperatively in addition to other antibiotics following surgery.

**Table 2:** Summary of patient-specific, operation-specific and practice-related factors investigated for association with surgical site infection (n = 861).

Characteristic	Summary (% infected)	Association with SSI^◼^
*Patient-specific risk factors*		
**Age (years)**		p = 0.99
<19.9	7 (14.3%)	
20.0–39.9	781 (14.3%)	
≥40	73 (15.1%)	
**ASA score**		p = 0.31
Missing	9	
I	291 (16.2%)	
II and III	561 (13.5%)	
**BMI prior to pregnancy (kg/m** ^**2**^ **)** ^**•**^		**p = 0.000** ^**♦**^
<36.0	833 (13.6%)	
≥36.0	28 (39.3%)	
**BMI prior to pregnancy (kg/m** ^**2**^ **)** ^**•**^		p = 0.02
Normal weight or underweight (<25)	386 (10.6%)	
Overweight (25–29.9)	308 (16.9%)	
Obese class-I (30.0–34.9)	125 (16.0%)	
Obese class-II (35–39.9)	30 (23.3%)	
Obese class-III ≥40	12 (33.3%)	
**Gestational age at delivery (week)**		**p = 0.012** ^**♦**^
Missing	1	
<39.9	753 (13.3%)	
>40	107 (22.4%)	
**Number of previous caesareans**		p = 0.72
0	338 (15.4%)	
1–2	425 (13.4%)	
≥3	98 (15.3%)	
**Smoking status**		p = 0.38
Current smoker	50 (18.0%)	
Previous smoker	39 (7.7%)	
Never smoked	772 (14.5%)	
**Weight gain during pregnancy** ^¶^		p = 0.63
<recommended weight gain range	239 (12.6%)	
Within recommended weight gain range	285 (15.1%)	
>recommended weight gain range	337 (15.1%)	
*Operation-specific risk factors*		
**Insertion of a surgical drain**		p = 0.19
Missing	2	
Yes	231 (16.9%)	
No	628 (13.4%)	
**Duration of admission (day)**		**p = 0.002** ^**♦**^
≤3.5	668 (12.4%)	
>3.5	193 (21.2%)	
**Duration of operation (min)** ^**+**^		p = 0.5
Infected wounds	50 (35,77.5)	
Clean wounds	50 (35,72.4)	
**Estimated blood loss during the operation (mL)**		p = 0.1
Missing	5	
<1000	799 (15.0%)	
≥1000	57 (7.0%)	
**Type of anaesthesia**		p = 0.22
Missing	4	
Epidural	51 (21.6%)	
General	92 (13.0%)	
Spinal	704 (13.8%)	
More than one type	10 (30.0%)	
**Type of surgery**		p = 0.35
Planned	498 (13.5%)	
Emergent	363 (15.7%)	
*Practice-specific risk factors*		
Administration of postoperative antibiotics*		p = 0.85
Yes	636 (13.7%)	
No	83 (14.5%)	
**Weight-adjusted cefazolin dose (mg/kg) (total number = 719)** ^+^		**p = 0.037** ^**♦**^
Infected wounds	18.7 (10.6, 28.6)	
Clean wounds	22.2 (11.1, 29.9)	

^■^Chi-square test was used for the analysis of categorical data, while a Mann-Whitney U-test was utilized for the analysis of continuous data. A p-value less than 0.05 was considered statistically significant. ASA score: American Society of Anaesthesiologists score. ^●^Height was imputed by 158.2 for 2.3% of eligible women. ^*^In patients receiving preoperative cefazolin. ^+^Continuous data were expressed as median (10^th^, 90^th^ percentiles). ^**♦**^Included in the multivariate analysis (p-value < 0.05). ^¶^Classified based on institute of medicine recommendations for weight gain during pregnancy taking into account BMI prior to pregnancy and the type of gestation (singleton vs. twin pregnancies). In the case of twin pregnancy, two cases of underweight women were treated as normal weight ladies. Two cases of triplet pregnancies were treated as twin pregnancies^11^. Term weight was calculated for 6.4% of eligible women by imputing weight gain with 13.5 kg.

During the regression analysis, several factors were found to be associated with infection; these factors included a higher BMI prior to pregnancy, lower weight-adjusted cefazolin dose, delivery beyond the 40^th^ week, and a hospital stay longer than 3.5 days. The study participants were under the care of ten consultants; when adjusting for this variable (categorized into two categories based on consultants’ obstetric experience), the results remained unchanged. The multiple regression analysis results are given in Table 3.

**Table 3:** Results of logistic regression analysis.

Risk factors	B	SE	p-value^*^	OR	95% CI	Reference category
Gestational age at delivery	0.81	0.29	0.005	2.24	1.3–3.92	<39.9 weeks
Weight-adjusted cefazolin dose	−0.034	0.016	0.035	0.967	0.94–0.99	—
Hospital stay	0.82	0.24	0.001	2.3	1.41–3.63	≤3.5 days
BMI	1.34	0.46	0.003	3.8	1.56–9.41	<36 kg/m^2^

^*^Multiple logistic regression, *p*-value < 0.05 was considered statistically significant.

Surgical site infection was coded 1, while 0 was assigned to patients who did not suffer from SSIs.

B, regression coefficient; SE, standard error associated with the coefficient B; OR, odds ratio; CI, confidence interval.

## Discussion

The present study determined the rate of SSIs during the 30 postoperative days and identified possible risk factors for the development of SSIs. The rate of surgical site infections detected in the present study was considerably high; approximately 3 out of 20 women undergoing a caesarean operation developed a SSI. Although this rate is high, it was within the range of reported rate of caesarean SSIs worldwide; the worldwide rates vary between low rates of infection of 2.1% in the United States^6^, high rates of 16% and 23.5% in Irish and Brazilian studies, respectively^5,7^, and to a rate as high as 48% in a resource-limited Tanzanian hospital^8^. Similar to other studies, staphylococcal microorganisms were the predominate type of infecting pathogens^9,10^, and other common pathogens included enterococcal bacteria. Enterococcal infections are not only common in caesareans^12^ but they are also thought to be the third most common cause of nosocomial infections globally, and special infection control measures are required in managing such infections^17^.

The preoperative dose of cefazolin was changed from 1 g during the initial period of the study to the ASHP recommended 2-g dose for non-obese women, although the 2-g dose was only marginally associated with a lower rate of SSIs compared with the 1-g dose, the higher term weight-adjusted cefazolin dose correlated with a lower risk for SSIs, as the odds ratio was 0.967; therefore, a 1-unit increase in the weight-adjusted cefazolin dose resulted in a 3.3% reduction in the risk of infection. Based on a median term weight of 80 kg, a change in dosing from 1 g to 2 g, in a typical pregnant woman, would result in an increase of 13 mg/kg units, which corresponds to a 35.6% reduction in the risk of wound infections. This result demonstrates the strong impact that a simple change in preoperative practice could have on the risk of SSIs. Furthermore, it is in line with the findings of a pharmacokinetic-pharmacodynamic simulation study that revealed that 2-g doses were more successful in maintaining blood levels at or above the target minimum inhibitory concentration of susceptible gram-positive bacteria during surgery^18^.

A recent study conducted by our group^19^ revealed that a major problem in the practice of surgical antibiotic prophylaxis at JUH is the prolonged administration of postoperative antibiotics. In light of the present results, an important modification to the current practice of surgical antibiotic prophylaxis administration at JUH would be the cessation of non-indicated postoperative antibiotic therapy, because we have not detected a difference in the rate of SSIs between women who received a single dose of preoperative cefazolin and those who continued to receive antibiotics postoperatively. This recommendation is supported by the ASHP guidelines and by several authors who have concluded that utilizing a single prophylactic dose (regardless of the type of antibiotic) was at least as effective, if not superior, to administering multiple doses in reducing infectious caesarean complications^20–22^. Obese women, on the other hand, constitute a special subpopulation that needs extensive research, particularly in light of the alarmingly high rates of obesity amongst Jordanian women and women of western countries^23,24^. New evidence arising from a recent randomized clinical trial indicates that, compared with placebo, obese women could benefit from a 2-day course of cephalexin and metronidazole^25^. Whether this approach is superior to a high single dose of cefazolin needs to be determined. Our results indicated a 4-fold increase in the risk of SSIs in obese women (BMI ≥36); the correlation between obesity and an increased risk of wound infection could be explained by a lower tissue concentration of the drug in obese patients, due to increases in the volume of distribution and the clearance of cephalosporins, necessitating an increase in the dose^26^.

A study conducted on 119,254 women who delivered following completion of the 37^th^ week found that both maternal and neonatal risk increase if delivery occurred at the 40^th^ week or beyond; such complications included chorioamnionitis, perineal laceration of 3^rd^ and 4^th^ degree, and non-reassuring foetal heart rate^27^. The present study adds a new complication for such types of deliveries, which is wound infection. Chaim *et al*.^28^ investigated the impact of having a caesarean at the gestational age of less than 37 weeks as opposed to greater than 37 weeks on the rate of SSIs, and found no significant difference in the rate of wound infections between the two groups. Similar results were obtained in our study when 37 weeks was used as a cutoff point (p = 0.6; chi-square test). However, when 40 weeks was used as a cutoff value, the risk of infection doubled in women delivering beyond 40 weeks.

The total duration of hospital stay in our population was relatively short (3.4 days on average). A prolonged postoperative stay was frequently related to patients’ attitude, as women refused to be discharged until their infant was released from the neonatal intensive care unit. Hospitalization longer than 3.5 days doubled the risk of subsequent SSIs development. Although a prolonged hospital stay was not associated with an increased risk of infection in gynaecological surgeries such as hysterectomy^29^, other researchers reported an increased risk of SSIs with prolonged total hospital stay and pre-hospital stay in general surgeries, probably due to an increase in the chance of antibiotic-resistant bacterial colonization with prolonged stays^30,31^.

Limitations of the study should be acknowledged. First, the follow-up period was only for the 30 postoperative days; therefore, the results of the present study are irrelevant to delayed infections developing beyond this time frame. Second, incomplete follow-up is a potential source of bias. However, because the baseline demographic, clinical and operation-related factors among the 312 excluded women and the 861 included women were similar, the impact of bias, although not completely eliminated, might be small. Third, the present study evaluated only SSIs, and other types of infectious caesarean complications such as urinary tract infections were not evaluated. Finally, the type of abdominal wound infection could not always be determined. An attempt was made to identify cases of organ space infections (endometritis) from patient-reported symptoms, as previously described by Wloch *et al*.^10^, however, this identification proved difficult, as some questions relating to this type of infection (Table S1: Q8–10) were confusing to women. As these questions could not be answered accurately, this section was removed from the final analysis. Nevertheless, in light of previous studies^10^ and the low readmission rate and mild course of symptoms usually reported by patients, it could be assumed that the vast majority of these infections were superficial abdominal wound infections.

In conclusion, the present study measured for the first time at a Jordanian hospital the rate of surgical site infection following caesarean operations during the initial 30 postoperative days and identified several risk factors for SSIs. Although some detected variables are non-modifiable, such as obesity, special measures can be taken at the clinical setting to provide optimal care and follow-up for this subpopulation. Other risk factors of SSIs detected in the present study can be easily modified; for instance, the authors recommend that caregivers should be instructed to administer 2-g cefazolin instead of 1-g in women weighing less than 120-kg. Hospital pharmacists should be given a central role in managing the practice of surgical antibiotic prophylaxis, as handling such a responsibility to pharmacists has recently been proven to be efficacious in enhancing optimal prophylactic antibiotic utilization^32,33^.

Another aspect of improvement is developing a post-discharge surveillance system. There is no ideal post-discharge surveillance system, but utilizing multiple approaches could help in identifying the largest possible proportion of these infections^34^. This could be accomplished by developing a special information technology infrastructure to create a hospital database that automatically links different possible sources of SSIs identification, including microbiological data, inpatient wards, outpatient clinics and coded transcribed phone interviews in addition to important patient-specific, procedure-specific and therapy-related factors that could influence SSIs. In this manner, hospitals could audit their infection rate regularly and take appropriate measures to optimize their clinical service.

## Electronic supplementary material

Supplementary information

## References

[1] Allegranzi B (2016). New WHO recommendations on preoperative measures for surgical site infection prevention: an evidence-based global perspective. Lancet Infect Dis..

[2] Zuarez-Easton S, Zafran N, Garmi G, Salim R (2017). Postcesarean wound infection: prevalence, impact, prevention, and management challenges. Int J Womens Health..

[3] Betrán AP (2016). The Increasing Trend in Caesarean Section Rates: Global, Regional and National Estimates: 1990–2014. PLoS One..

[4] Al Rifai R (2014). Rising cesarean deliveries among apparently low-risk mothers at university teaching hospitals in Jordan: analysis of population survey data, 2002–2012. Glob Heal Sci Pract..

[5] Del Monte MCC, Pinto Neto AM (2010). Postdischarge surveillance following cesarean section: The incidence of surgical site infection and associated factors. Am J Infect Control..

[6] Hickson E, Harris J, Brett D (2015). A Journey to Zero: Reduction of Post-Operative Cesarean Surgical Site Infections over a Five-Year Period. Surg Infect (Larchmt)..

[7] Corcoran S (2013). Surgical site infection after cesarean section: Implementing 3 changes to improve the quality of patient care. Am J Infect Control..

[8] De Nardo P (2016). Post-caesarean section surgical site infections at a Tanzanian tertiary hospital: a prospective observational study. J Hosp Infect..

[9] Kaplan NM, Smadi AA, Al-Taani MI, El-Qudah MA (2003). Microbiology of wound infection after caesarean section in a Jordanian hospital. East Mediterr Health J..

[10] Wloch C (2012). Risk factors for surgical site infection following caesarean section in England: results from a multicentre cohort study. BJOG..

[11] American College of Obstetricians and Gynecologists. Weight Gain During Pregnancy. ACOG committee opinion no. 548. O*bs Gynecol*. 1**21**, 210–2 (2013). 10.1097/01.aog.0000425668.87506.4c 23262962

[12] Bratzler DW (2013). Clinical practice guidelines for antimicrobial prophylaxis in surgery. Am J Heal Pharm..

[13] Centers for Disease Control and Prevention. Surgical SiteInfection (SSI) Event. http://www.cdc.gov/nhsn/PDFs/pscManual/9pscSSIcurrent.pdf?agree=yes&nex=Accept. 2014 Jan. Last accessed April 2017.

[14] Horan TC (2008). CDC/NHSN surveillance definition of health care-associated infection and criteria for specific types of infections in the acute care setting. Am J Infect Control..

[15] Vittinghoff E, McCulloch CE (2007). Relaxing the Rule of Ten Events per Variable in Logistic and Cox Regression. Am J Epidemiol..

[16] Ploeg Tvander (2014). Modern modelling techniques are data hungry: a simulation study for predicting dichotomous endpoints. BMC Med Res Methodol..

[17] Rajkumari N, Mathur P, Misra MC (2014). Soft Tissue and Wound Infections Due to Enterococcus spp. Among Hospitalized Trauma Patients in a Developing Country. J Glob Infect Dis..

[18] Elkomy MH (2014). Pharmacokinetics of prophylactic cefazolin in parturients undergoing cesarean delivery. Antimicrob Agents Chemother..

[19] Abdel Jalil, M. H. *et al*. Noncompliance with surgical antimicrobial prophylaxis guidelines: A Jordanian experience in cesarean deliveries. A*m J Infect Control* [Epub ahead print]. (2017). 10.1016/j.ajic.2017.06.033 28800838

[20] Shakya A, Sharma J (2010). Comparison of single versus multiple doses of antibiotic prophylaxis in reducing post-elective Caesarean section infectious morbidity. Kathmandu Univ Med J..

[21] Alekwe LO, Kuti O, Orji EO, Ogunniyi SO (2008). Comparison of ceftriaxone versus triple drug regimen in the prevention of cesarean section infectious morbidities. J Matern Neonatal Med..

[22] Lyimo FM, Massinde AN, Kidenya BR, Konje ET, Mshana SE (2013). Single dose of gentamicin in combination with metronidazole versus multiple doses for prevention of post-caesarean infection at Bugando Medical Centre in Mwanza, Tanzania: a randomized, equivalence, controlled trial. BMC Pregnancy Childbirth..

[23] Al Nsour M, Al Kayyali G, Naffa S (2013). Overweight and obesity among Jordanian women and their social determinants. East Mediterr Health J..

[24] Cuschieri S, Mamo J (2016). Getting to grips with the obesity epidemic in Europe. SAGE Open Med..

[25] Valent AM (2017). Post-operative prophylactic oral antibiotics for preventing surgical site infections in obese women undergoing cesarean deliveries: A randomized controlled trial. Am J Obstet Gynecol..

[26] American College of Obstetricians and Gynecologists. Practice Bulletin No. 120: Use of Prophylactic Antibiotics in Labor and Delivery. O*bstet Gynecol*. 1**17**, 1472–1483 (2011). 10.1097/AOG.0b013e3182238c31 21606770

[27] Caughey AB, Stotland NE, Washington AE, Escobar GJ (2007). Maternal and obstetric complications of pregnancy are associated with increasing gestational age at term. Am J Obstet Gynecol..

[28] Chaim W, Bashiri A, Bar-David J, Shoham-Vardi I, Mazor M (2000). Prevalence and clinical significance of postpartum endometritis and wound infection. Infect Dis Obstet Gynecol..

[29] Pop-Vicas A, Musuuza JS, Schmitz M, Al-Niaimi A, Safdar N (2017). Incidence and risk factors for surgical site infection post-hysterectomy in a tertiary care center. Am J Infect Control..

[30] Isik O, Kaya E, Dundar HZ, Sarkut P (2015). Surgical Site Infection: Re-assessment of the Risk Factors. Chirurgia (Bucur)..

[31] Akhter MSJ, Verma R, Madhukar KP, Vaishampayan AR, Unadkat PC (2016). Incidence of surgical site infection in postoperative patients at a tertiary care centre in India. J Wound Care..

[32] Zhou L, Ma J, Gao J, Chen S, Bao J (2016). Optimizing Prophylactic Antibiotic Practice for Cardiothoracic Surgery by Pharmacists’ Effects. Medicine (Baltimore)..

[33] Wang J (2015). Impact of pharmacist interventions on rational prophylactic antibiotic use and cost saving in elective cesarean section. Int J Clin Pharmacol Ther..

[34] Halwani MA, Turnbull AE, Harris M, Witter F, Perl TM (2016). Postdischarge surveillance for infection following cesarean section: A prospective cohort study comparing methodologies. Am J Infect Control..

