# Supplementary material for: Surgical site infections following caesarean operations at a Jordanian teaching hospital: Frequency and implicated factors
Source: Sci Rep. 2017 Sep 22;7:12210. doi: 10.1038/s41598-017-12431-2 (PMC5610177; doi:10.1038/s41598-017-12431-2)
Supplement: Supplementary file 1 — Supplementary information [file 41598_2017_12431_MOESM1_ESM.pdf]

Surgical site infections following caesarean operations at a Jordanian teaching hospital: Frequency and implicated factors

Mariam Hantash Abdel Jalil[1], **Khawla Abu Hammour**[1], **Mervat Alsous**[2], **Wedad Awad**[1], **Rand Hadadden**[1], **Faris Bakri**[3,4], **Kamil Fram**[5]

[1]Department of Biopharmaceutics and Clinical Pharmacy, Faculty of Pharmacy, University of Jordan, Amman-Jordan
[2] Department of Clinical Pharmacy and Therapeutics, Faculty of Pharmacy, Applied Science Private University, Amman-Jordan
[3]Department of Medicine-Division of Infectious Diseases, University of Jordan, Jordan University Hospital, Amman -Jordan
[4]Infectious Diseases and Vaccine Centre, University of Jordan, Amman -Jordan
[5]Department of Obstetrics and Gynaecology, University of Jordan, Jordan University Hospital, Amman -Jordan

SUPPLEMENTARY INFORMATION

Table S1: Summary of questions asked during the telephone interview*

**1. Did you suffer from elevated body temperature (greater than 38°)?** Yes/ No<br>If yes, specify the reason, onset and duration of fever.<br>**2. Have you had any of the following problems with your wound?** Yes/ No<br>Redness<br>Heat<br>Swelling<br>Pain<br>If yes, specify onset and duration of symptoms.<br>**3. Did you notice any discharge from your wound?** Yes/ No<br>If yes, how was it?<br>Yellow discharge, bloody discharge, other (please specify)<br>When did it start and how long did it last?<br>**4. Have you been told by your doctor that your wound was infected?** Yes/ No<br>If yes, specify when.<br>**5. Did your wound re-open?** Yes/ No<br>If yes, specify when and measures taken.<br>**6. Was your wound deliberately opened by your physician?** Yes/ No<br>If yes, specify when and why was the wound opened<br>**7. Have your doctor prescribed antibiotics for the purpose of wound infection?** Yes/No<br>If yes, specify when<br>**8. Did you suffer from uterine tenderness?** Yes/No<br>If yes, specify when<br>**9. Did you suffer from abdominal pain?** Yes/No<br>If yes, specify when<br>**10. Did you notice a purulent uterine discharge?** Yes/No<br>If yes, specify when
*These questions were asked in the Arabic language, all questions were related to the initial 30 days following the operation.

Table S2: Criteria for identifying surgical site infections (SSIs) based on patient-reported symptoms and antibiotic usage (Wloch *et al*[1])

**Criterion 1**	The patient was prescribed antibiotics for the purpose of treating a SSI and the wound was discharging pus
**Criterion 2**	The patient suffered from at least two of the following clinical symptoms: pain, heat, redness or swelling, in addition to wound dehiscence or deliberate opening of the wound by the surgeon or attending physician
**Criterion 3**	The patient was prescribed antibiotics for the purpose of treating a SSI and suffered from at least two of the following clinical symptoms: pain, heat, redness or swelling

Table S3: Patient-specific, operation-specific and practice-related factors investigated for association with surgical site infections in included and excluded patients

Characteristic	All participants	Included participants		Excluded participants♦	Statistical significance■ (excluded vs. included)
	Summary	Summary (% infected)	Association with SSI■	Summary	
**Number**	1173	861		312	
Patient-specific risk factors					
**Age (years)**			p=0.99		p=0.49
<19.9	12	7 (14.3%)		5	
20-39.9	1062	781 (14.3%)		281	
≥40	99	73 (15.1%)		26	
**ASA score**			p=0.31		p=0.65
Missing	12	9		3	
I	401	291 (16.2%)		110	
II and III	760	561 (13.5%)		199	
**BMI prior to pregnancy (kg/m^2)** •			**p=0.000****		p=0.97
<36.0	1135	833 (13.6%)		302	
≥36.0	38	28 (39.3%)		10	
**BMI prior to pregnancy (kg/m^2)** •			p=0.02		p=0.42
Normal weight or underweight (<25)	531	386 (10.6%)		145	
Overweight (25-29.9)	428	308 (16.9%)		120	
Obese class-I (30.0-34.9)	159	125 (16.0%)		34	
Obese class-II (35-39.9)	41	30 (23.3%)		11	
Obese class-III ≥40	14	12 (33.3%)		2	
**Gestational age at delivery (week)**			**p=0.012****		p=0.41
Missing	4	1		3	
<39.9	1029	753 (13.3%)		276	
>40.0	140	107 (22.4%)		33	
**Number of previous caesareans**			p=0.72		p=0.22
0	444	338 (15.4%)		106	
1-2	596	425 (13.4%)		171	
≥3	133	98 (15.3%)		35	
**Parity**			p=0.31		p=0.86
Nulliparous	249	185 (17.8%)		64	
Monoparous	301	216 (15.3%)		85	
Multiparous	529	389 (13.1%)		140	
Grand-multiparous	94	71 (9.9%)		23	
**Smoking status**			p=0.38		p=0.41
Current smoker	62	50 (18.0%)		12	
Previous smoker	53	39 (7.7%)		14	
Never smoked	1058	772 (14.5%)		286	
**Weight gain during pregnancy****			p=0.63		p=0.95
< Recommended weight gain range	323	239 (12.6%)		84	
Within recommended weight gain range	388	285 (15.1%)		103	
> Recommended weight gain range	462	337 (15.1%)		125	
Operation-specific risk factors					
**Insertion of a surgical drain**			p=0.19		p=0.34
Missing	3	2		1	
Yes	306	231 (16.9%)		75	
No	864	628 (13.4%)		236	
**Duration of admission (day)**			**p=0.002****		p=0.73
≤3.5	913	668 (12.4%)		245	
>3.5	260	193 (21.2%)		67	
**Duration of operation (min)** ***			p=0.5	50 (35,74.1)	p=0.21
Infected wounds	124¶	50 (35,77.5)			
Clean wounds	737¶	50 (35,72.4)			
**Estimated blood loss during the operation (mL)**			p=0.1		p=0.64
Missing	11	5		6	
<1000	1087	799 (15.0%)		288	
≥1000	75	57 (7.0%)		18	
**Time of incision**			p=0.86		p=0.90
Shift A: 08:00-16:00	834	615 (14.1%)		219	
Shift B: 16:00-00:00	236	172 (15.7%)		64	
Shift C: 00:00-8:00	103	74 (13.5%)		29	
**Type of anaesthesia**			p=0.22		p=0.78
Missing	7	4		3	
Epidural	68	51 (21.6%)		17	
General	124	92 (13.0%)		32	
Spinal	958	704 (13.8%)		254	
More than one type	16	10 (30.0%)		6	
**Type of surgery**			p=0.35		p=0.26
Planned	690	498 (13.5%)		192	
Emergent	483	363 (15.7%)		120	
**Skin suture material**			p=0.93		p=0.41
Missing data or used stapler	108	77		31	
Braided thread	250	179 (14.0%)		71	
Monofilament thread	815	605 (14.2%)		210	

Characteristic	All participants	Included participants		Excluded participants[♦]	Statistical significance[■] (excluded vs. included)
	Summary	Summary (% infected)	Association with SSI[■]	Summary	
**Administration of blood products during admission**			p=0.94		p=0.20
Yes	124	85 (14.1%)		39	
No	1049	776 (14.4%)		273	
Practice-specific risk factors					
**Administration of post-operative antibiotics**[⁋]			p=0.85		p=0.67
Yes	866	636 (13.7%)		230	
No	116	83 (14.5%)		33	
**Weight adjusted cefazolin dose (mg/kg) (total number=982)** [***]			**p=0.037**[*]	22.7 (11.5,30.3)	p=0.33
Infected wounds	99[¶]	18.7 (10.6, 28.6)			
Clean wounds	620[¶]	22.2 (11.1, 29.9)			

[■]Chi-square test was used for the analysis of categorical data, while a Mann-Whitney U-test was utilized for the analysis of continuous data. A p-value less than 0.05 was considered statistically significant.
[♦]Patients were excluded from the SSI analysis due to lack of follow-up or incomplete follow-up.
ASA score: American Society of Anaesthesiologists score.
[●] Height was imputed by 158.2 for 2.3% of eligible women.
[*] Included in the multivariate analysis (p-value < 0.05).
[**]Classified based on institute of medicine recommendations for weight gain during pregnancy taking into account BMI prior pregnancy and the type of gestation (singleton vs. twin pregnancies). In the case of twin pregnancy, two cases of underweight women were treated as normal weight ladies. Two cases of triplet pregnancies were treated as twin pregnancies[2]. Term weight was calculated for 6.4% of eligible women by imputing weight gain with 13.5 kg.
[***]Continuous data were expressed as median (10th, 90th percentiles).
[¶]Number represents included patients only.
[⁋]In patients receiving preoperative cefazolin.

References:

1 Wloch, C., et al. Risk factors for surgical site infection following caesarean section in England: results from a multicentre cohort study. *BJOG*. **119**, 1324–33 (2012).

2 American College of Obstetricians and Gynecologists. Weight Gain During Pregnancy. ACOG committee opinion no. 548. *Obs Gynecol*. **121**, 210–2 (2013).
